# Management of Arrhythmias and Conduction Disorders in Amyloid Cardiomyopathy

**DOI:** 10.3390/jcm13113088

**Published:** 2024-05-24

**Authors:** Katarzyna Holcman, Andrzej Ząbek, Krzysztof Boczar, Piotr Podolec, Magdalena Kostkiewicz

**Affiliations:** 1 Department of Nuclear Medicine, John Paul II Hospital, 31-202 Krakow, Poland; kostkiewiczmagda@gmail.com; 2 Department of Cardiac and Vascular Diseases, Jagiellonian University Medical College, John Paul II Hospital, 31-202 Krakow, Poland; ppodolec@interia.pl; 3 Department of Electrocardiology, Jagiellonian University Medical College, John Paul II Hospital, 31-202 Krakow, Poland; andrzej_j_z@poczta.onet.pl (A.Z.); krzysiek.boczar@gmail.com (K.B.)

**Keywords:** amyloidosis, amyloid cardiomyopathy, ATTR, AL

## Abstract

Cardiac amyloidosis, a condition characterized by abnormal protein deposition in the heart, leads to restrictive cardiomyopathy and is notably associated with an increased risk of arrhythmias and conduction disorders. This article reviews the current understanding and management strategies for these cardiac complications, with a focus on recent advancements and clinical challenges. The prevalence and impact of atrial arrhythmias, particularly atrial fibrillation, are examined, along with considerations for stroke risk and anticoagulation therapy. The article also addresses the complexities of managing rate and rhythm control, outlining the utility and limitations of pharmacological agents and interventions such as catheter ablation. Furthermore, it reviews the challenges in the treatment of ventricular arrhythmias, including the contentious use of implantable cardioverter-defibrillators for primary and secondary prevention. Individualized approaches, considering the unique characteristics of cardiac amyloidosis, are paramount. Continuous research and clinical exploration are essential to refine treatment strategies and improve outcomes in this challenging patient population.

## 1. Introduction

**Figure 1:**
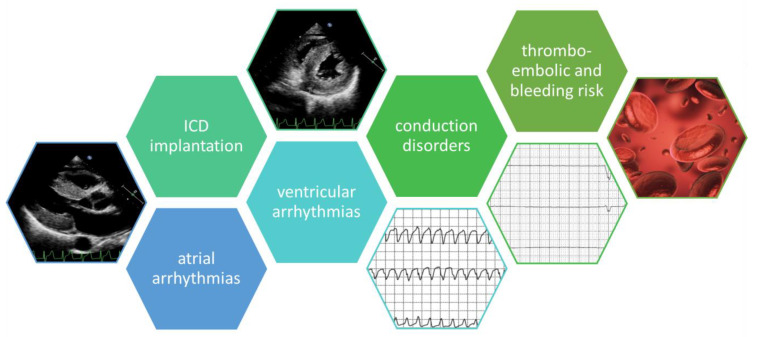
Clinical aspects of arrhythmias and conduction disorders, and echocardiographic findings in amyloid cardiomyopathy (descriptions of panels from the left—transthoracic echoacardiography, parasternal long-axis projection, morphological features of transthyretin amyloidosis of the heart; transthoracic echoacardiography, parasternal short-axis view, visible concentric thickening of the granular muscle of the left ventricle; resting electrocardiogram, ventricular tachycardia in a patient with amyloid cardiomyoapathy; Holter monitoring, visible pause; thromboembolic risk).

Amyloidosis, a complex systemic disorder characterized by abnormal protein fibril deposition, significantly impacts the heart, leading to restrictive cardiomyopathy [1,2]. The prevalence of this condition has seen a notable increase, challenging previous estimates and necessitating a reevaluation of disease burden (Table 1) [3]. Cardiac amyloidosis, marked by transthyretin or immunoglobulin light-chain deposits, is increasingly recognized as a crucial contributor to heart failure [4]. Recent epidemiological trends indicate a surge in diagnoses of wild-type transthyretin amyloid cardiomyopathy (wtATTR-CM), particularly in individuals over 80 [5]. The prevalence of hereditary transthyretin amyloid cardiomyopathy (hATTR-CM) varies with specific gene variants [6]. Additionally, the plasma cell proliferative disorder, light-chain amyloidosis (AL), adds to the complexity of cardiac amyloidosis, with prevalence estimates ranging from 15.5 to 40.5 cases per million [7]. Beyond heart failure, cardiac amyloidosis heightens susceptibility to arrhythmias, encompassing atrial fibrillation (AF), supraventricular tachycardias, ventricular arrhythmias, and intrinsic conduction disease [8]. These arrhythmias, often symptomatic and poorly tolerated, demand expedited and individualized treatment [9]. Despite the commonality of sudden death, ventricular arrhythmia, and conduction disease, the timing of interventions like implantable cardioverter-defibrillator (ICD) or pacemaker implantation remains debatable (Figure 1) [10].

## 2. Atrial Arrhythmias in Cardiac Amyloidosis

### 2.1. Underlying Pathomechanisms and Epidemiology

Arrhythmias in cardiac amyloidosis arise from the intricate interplay of amyloid fibril deposition, resulting in myocardial wall thickening, impaired relaxation, and restrictive filling [11]. The pathogenesis involves elevated filling pressure inducing atrial dilation, fostering the development of AF and other atrial arrhythmias [12]. Atrial amyloid deposition contributes to myocardial fibrosis and structural remodeling, further predisposing patients to AF [13].

The prevalence of atrial arrhythmias in cardiac amyloidosis surpasses that of the general population [14]. Notably, wtATTR-CM carries the highest risk of comorbid AF, with prevalence estimates reaching up to 44% [15]. This emphasizes the significant impact of wtATTR-CM on the atrial substrate, leading to a higher propensity for arrhythmias [16]. Ongoing amyloid deposition within atrial tissue electro-anatomically disrupts homogeneous electrical conduction; furthermore, the direct toxic effect of amyloid fibrils on cardiomyocytes results in fibrosis and oxidative stress, leading to AF development [16].

In addition to the increased prevalence associated with wtATTR-CM, hATTR-CM exhibits variable prevalence rates based on specific gene variants [17]. Understanding the genetic basis of hATTR-CM becomes pivotal in gauging the risk and implementing targeted management strategies (Table 2) [18]. AL subtype, characterized by a plasma cell proliferative disorder, also contributes to the overall prevalence of atrial arrhythmias in cardiac amyloidosis [19,20]. Despite the commonality of atrial arrhythmias, the presence or specific subtype of AF in cardiac amyloidosis does not appear to significantly impact overall survival [21]. This intriguing observation prompts further investigation into the nuanced relationship between atrial arrhythmias and the overall prognosis of patients with cardiac amyloidosis [22].

### 2.2. Thromboembolic Risk

Cardiac amyloidosis, characterized by the extracellular deposition of amyloid fibrils in the myocardium, significantly alters the dynamics of blood flow within the heart chambers [23]. This structural remodeling, coupled with impaired relaxation and restrictive filling, creates a milieu conducive to intracardiac thrombus formation [24]. The risk of thrombus formation is particularly heightened in the atria, where amyloid deposition and dilation contribute to stasis of blood flow [25]. Atrial arrhythmias, including AF, are prevalent in cardiac amyloidosis and further exacerbate the risk of stroke [26]. The irregular and often rapid atrial contractions in AF can lead to blood pooling in the atria, increasing the likelihood of thrombus formation [27]. Additionally, the compromised atrial mechanical function in cardiac amyloidosis contributes to ineffective atrial contraction, promoting stasis and thromboembolism [28]. The assessment of thrombotic risk in cardiac amyloidosis necessitates a nuanced approach, recognizing the unique hemodynamic challenges posed by this condition [29]. Traditional risk stratification tools, such as the CHA2DS2-VASc score commonly used in AF, may not fully capture the intricacies of thrombotic risk in cardiac amyloidosis [30]. Transesophageal echocardiogram (TEE) emerges as a valuable tool for assessing intracardiac thrombus, especially before procedures like direct current cardioversion (DCCV) [31]. The sensitivity of TEE in detecting thrombus formation provides critical information for guiding anticoagulation decisions in patients with cardiac amyloidosis and atrial arrhythmias [32].

The management of anticoagulation in cardiac amyloidosis requires a careful balance between preventing thromboembolic events and avoiding potential complications, such as bleeding [33]. Warfarin, a vitamin K antagonist, has historically been the mainstay of anticoagulation therapy in AF [34]. However, the use of novel oral anticoagulants (NOACs) is gaining attention due to their predictable pharmacokinetics and reduced monitoring requirements [35]. The choice between warfarin and NOACs should be individualized based on patient factors, including renal function, drug interactions, and the potential for compliance [36]. The increased prevalence of renal impairment in cardiac amyloidosis underscores the importance of assessing renal function when selecting anticoagulation therapy [37].

Assessing bleeding risk is crucial in determining the appropriateness of anticoagulation in patients with cardiac amyloidosis [38]. The underlying amyloid deposition and associated vascular fragility may increase the risk of bleeding events [39]. Therefore, a comprehensive evaluation of both thrombotic and bleeding risks is essential for informed decision-making [40].

### 2.3. Rate Control

Understanding hemodynamics in cardiac amyloidosis is vital for managing atrial arrhythmias effectively [41]. The restrictive physiology inherent in cardiac amyloidosis necessitates a cautious approach to rate control, as conventional medications may exacerbate heart failure [42]. Amiodarone emerges as a well-tolerated antiarrhythmic in this context, though the optimal strategy for rate control warrants further investigation [43]. Pharmacological agents play a central role in achieving rate control in atrial arrhythmias [44]. Beta-blockers, calcium channel blockers, and digoxin are among the commonly used medications in the general population [45]. However, in the context of cardiac amyloidosis, factors such as drug interactions, compromised renal function, and potential side effects need careful consideration [46].

- Beta-blockers: These agents, such as metoprolol and carvedilol, are often used as first-line therapy. They exert their effects by blocking the beta-adrenergic receptors, thereby reducing the heart rate and myocardial oxygen demand. However, caution is required in patients with cardiac amyloidosis, due to potential exacerbation of heart failure and conduction disturbances.
- Calcium channel blockers: Verapamil and diltiazem are calcium channel blockers commonly used for rate control. They act by inhibiting calcium influx into the cells, leading to decreased cardiac contractility and heart rate. Since this group causes hypotension and conduction abnormalities, they are contraindicated in cardiac amyloidosis.
- Digoxin: While digitalis glycosides like digoxin can be effective in controlling heart rate, their use in cardiac amyloidosis is contraindicated. The risk of toxicity is elevated due to altered drug metabolism and potential drug interactions.

### 2.4. Rhythm Control

Contrary to the potential risks associated with conventional medications, antiarrhythmics, particularly amiodarone, find favor in managing atrial arrhythmias in cardiac amyloidosis [47]. Despite limited evidence questioning the mortality benefit of rhythm control, the unique pathophysiological characteristics of cardiac amyloidosis underscore the need for tailored approaches to rhythm management, especially in asymptomatic individuals [48]. The selection of medications requires careful consideration of the underlying disease, potential drug interactions, and the overall hemodynamic status of the patient.

- Amiodarone: This class III antiarrhythmic agent is often considered a first-line therapy in cardiac amyloidosis due to its broad spectrum of activity. However, the risk of adverse effects, including pulmonary toxicity and liver dysfunction, necessitates close monitoring.
- Dofetilide: Class III antiarrhythmics like dofetilide may be used to restore and maintain sinus rhythm. However, caution is advised in patients with impaired renal function, which is not uncommon in cardiac amyloidosis.
- Flecainide and propafenone: These class IC antiarrhythmics may be considered in certain cases, but their use requires careful evaluation of ventricular function and the absence of structural heart disease.
- Sotalol: A class III antiarrhythmic with both beta-blocking and antiarrhythmic properties, sotalol may be employed cautiously, particularly in patients without significant structural heart disease.

### 2.5. Direct Current Cardioversion and Catheter Ablation

While direct current cardioversion (DCCV) may present an attractive option for managing symptomatic atrial tachyarrhythmias, studies report variable success rates and recurrence risks in the context of cardiac amyloidosis [49]. Catheter ablation, though constrained by limited available data, shows promise as an intervention. However, the outcomes of catheter ablation in cardiac amyloidosis remain an area of ongoing research, highlighting the need for further investigation into the effectiveness of these interventions in this unique patient population [50].

Candidates for DCCV are carefully selected based on factors such as the duration of atrial arrhythmia, symptoms, and overall cardiovascular health. Pre-procedure evaluation includes a thorough assessment of the patient’s medical history, medications, and the presence of any reversible causes of atrial arrhythmias. DCCV is highly effective in restoring sinus rhythm, especially in cases of recent onset AF. However, the long-term success of DCCV in maintaining normal rhythm can be influenced by factors such as the underlying cause of arrhythmia and the presence of structural heart disease. The recurrence of atrial arrhythmias after DCCV is not uncommon. Therefore, post-procedural management often involves a combination of antiarrhythmic medications and ongoing monitoring.

Catheter ablation, a procedure aimed at eliminating the abnormal electrical pathways responsible for atrial arrhythmias, is gaining attention in the context of cardiac amyloidosis. Patient selection for catheter ablation involves a comprehensive evaluation of the type and duration of atrial arrhythmia, the presence of structural heart disease, and the overall clinical status of the patient. Pre-procedural imaging, including advanced cardiac imaging techniques like cardiac magnetic resonance imaging (MRI), may be employed to assess the extent of amyloid deposition and guide the ablation strategy. The presence of amyloid deposits can complicate catheter ablation by altering tissue consistency and increasing the risk of procedural complications. Careful consideration is given to the choice of energy source and ablation strategy to balance the need for effective lesion creation with the avoidance of collateral damage. Success rates of catheter ablation in cardiac amyloidosis vary, and the procedure may need to be repeated in some cases. Donnellan et al. (2020) found that, during a mean follow-up of 39 months, the recurrence rate of AF after ablation in patients with ATTR amyloidosis was 58%; however, there was a marked reduction in hospitalization rates for AF and heart failure, suggesting a clinical benefit [15]. Post-ablation care involves close monitoring for recurrence of atrial arrhythmias, and the continuation of antiarrhythmic medications may be necessary.

The role of AF ablation in patients with heart failure, including those with end-stage heart failure, is critically important. A meta-analysis comparing catheter ablation and medical therapy for atrial fibrillation in heart failure patients demonstrated significant benefits of ablation, in terms of all-cause mortality and improvements in left ventricular ejection fraction and quality of life [51]. Various catheter ablation approaches have been developed to treat arrhythmia recurrence in patients with durable pulmonary vein isolation, highlighting the efficacy of repeat ablation in improving clinical outcomes [52].

## 3. Overview of the Conduction Anomalies in Cardiac Amyloidosis

A foundational comprehension of the cardiac conduction system is critical for understanding conduction anomalies in cardiac amyloidosis [51]. Amyloid deposits exert infiltrative effects on the conduction tissues of the heart, disrupting the conventional architecture of the conduction system [53,54]. Diverse conduction abnormalities manifest in cardiac amyloidosis, encompassing the following:

- Sinus node dysfunction: amyloid infiltration in the SA node precipitates sinus bradycardia, sinus arrest, or sinoatrial block.
- Atrioventricular (AV) block: amyloid involvement in the AV node yields degrees of block, ranging from first-degree to complete heart block.
- Intraventricular conduction delays: amyloid deposits within the ventricles induce delays in conduction pathways, perturbing the coordination of ventricular contraction.

Clinical presentations of conduction abnormalities in cardiac amyloidosis exhibit wide variation. Patients may either remain asymptomatic or experience manifestations such as dizziness, syncope, or palpitations. The severity of symptoms correlates proportionately with the degree of conduction abnormality. The diagnosis of conduction disease in cardiac amyloidosis necessitates a comprehensive approach, incorporating clinical assessment, electrocardiography (ECG), and advanced imaging techniques when required. ECG findings may reveal characteristic abnormalities, including prolonged PR intervals, AF, or complete heart block.

The effectual management of conduction disease in cardiac amyloidosis mandates a multidisciplinary approach. Depending on the severity of conduction abnormalities and associated symptoms, interventions may encompass temporary or permanent pacemaker implantation for symptomatic bradycardia or heart block, pharmacological interventions tailored to address specific conduction anomalies (Table 3). Overall, patients with wild-type transthyretin cardiac amyloidosis (ATTRwt) may be more susceptible to AV involvement, compared to AL [9]. Moreover, patients with ATTR more frequently require pacemaker implantation [9]. Indeed, prophylactic pacemaker implantation may further reduce the burden of major cardiovascular events [50]. The presence and severity of conduction abnormalities in cardiac amyloidosis hold prognostic significance [53,54]. Advanced conduction aberrations may contribute substantively to augmented morbidity and mortality, underscoring the criticality of timely diagnosis and intervention. Further research will show the effects of resynchronization therapy in this group of patients. In patients with ATTR, frequent right ventricular pacing has been shown to be associated with worsening mitral regurgitation, ejection fraction, and heart failure symptoms, compared to the effects of biventricular pacing [49].

## 4. Ventricular Arrhythmias in Cardiac Amyloidosis

Cardiac amyloidosis introduces a complex landscape of arrhythmogenic potential, including ventricular arrhythmias [1,55,56,57,58,59,60]. Studies have shown that up to one-half of patients with cardiac amyloidosis experience sudden death. However, the use of ICDs for primary prevention in this context has not garnered robust support from expert guidelines, despite a high arrythmia burden in this population [56].

### 4.1. ICD Recommendations

Historically, sudden death in cardiac amyloidosis has been attributed to electromechanical dissociation leading to pulseless electrical activity rather than lethal ventricular arrhythmias. Concerns have been raised about a potentially higher defibrillation threshold in cardiac amyloidosis, possibly making it refractory to ICD therapy. The historically poor prognosis and life expectancy in this population have contributed to the cautious approach towards ICD placement. The notion that electromechanical dissociation is the primary reason for sudden death may lack robust support. Studies often cited to suggest this are based on small cohorts with untreated disease and high mortality rates, potentially not representative of the entire population.

The European Society of Cardiology (ESC) Guidelines acknowledge insufficient data for providing recommendations on ICD use for primary prevention in cardiac amyloidosis [61]. For secondary prevention, both the ESC and the 2017 American Heart Association/American College of Cardiology/Heart Rhythm Society guidelines advocate individualized decision-making [62]. The latter recommends consideration for those with ventricular arrhythmias causing hemodynamic instability and an expected survival of more than one year with good functional status. The 2022 ESC Guidelines underline that the benefit of primary prevention ICD implantation in patients with cardiac amyloidosis is uncertain [61]. Currently, an ICD should be considered in patients with hemodynamically not-tolerated VT after a careful discussion of the competing risks of non-arrhythmic death and non-cardiac death [61].

Society guidelines, based on case reports and observational studies, highlight the limited data on the efficacy of ICDs in primary or secondary prevention in cardiac amyloidosis. Studies by Lin et al. and Varr et al. report varying rates of appropriate ICD therapies for sustained ventricular tachyarrhythmias, with no clear association with improved mortality [49,58]. Recent studies have consistently demonstrated challenges in achieving successful ICD therapies for primary prevention [56]. The clinical implications of ICD use in cardiac amyloidosis demand careful consideration, especially in the context of limited data and varying outcomes reported in recent studies and disease-modifying therapies in specific subpopulations [1,58,59,60]. Ongoing research is crucial to elucidate the specific factors influencing ICD efficacy in this population and to guide more precise recommendations for primary and secondary prevention.

The decision to employ ICDs for primary and secondary prevention in cardiac amyloidosis remains a nuanced and evolving aspect of clinical management. The interplay of disease-specific factors, limited data, and varied outcomes underscores the need for individualized decision-making and continuous exploration of therapeutic strategies (Table 4).

### 4.2. Pharmacotherapy

Pharmacotherapy, including amiodarone, beta-blockers, mexiletine, and sotalol, plays a significant role in managing ventricular arrhythmias in cardiac amyloidosis [57]. Amiodarone, a class III antiarrhythmic agent, stands out as a prominent pharmacotherapeutic option. Its broad-spectrum activity makes it a viable choice for managing ventricular arrhythmias in cardiac amyloidosis. However, vigilant monitoring is imperative due to potential adverse effects, including pulmonary toxicity and liver dysfunction. Beta-blockers are often considered first-line therapy for ventricular arrhythmias. However, caution is warranted in patients with cardiac amyloidosis to prevent exacerbation of heart failure and conduction disturbances. Sodium channel blockers like mexiletine may be employed, particularly in cases where other antiarrhythmics are contraindicated or not well-tolerated. The use of mexiletine should be judicious, considering potential side effects and drug interactions. Sotalol, a class III antiarrhythmic with beta-blocking properties, may be cautiously employed, especially in patients without significant structural heart disease.

### 4.3. Catheter Ablation for Ventricular Arrhythmias

Patient selection for catheter ablation involves a comprehensive evaluation of the type and frequency of ventricular arrhythmias, the presence of structural heart disease, and the overall clinical status of the patient. Advanced imaging techniques, including cardiac MRI, may be instrumental in assessing the extent of amyloid deposition and guiding the ablation strategy.

The infiltrative nature of amyloid deposits can complicate catheter ablation, altering tissue consistency and increasing the risk of procedural complications. Careful consideration of the choice of energy source and ablation strategy is crucial to balance effective lesion creation with the avoidance of collateral damage. Success rates of catheter ablation in cardiac amyloidosis may vary, and the procedure might need repetition in some cases [56]. Post-ablation care involves close monitoring for recurrence of ventricular arrhythmias, and the continuation of antiarrhythmic medications may be necessary.

While the role of catheter ablation for the treatment of atrial arrhythmias is progressing, with the current literature indicating potential benefits in terms of recurrence rates and overall survival, there is a paucity of data on its application in ventricular arrhythmias [38]. Apart from case reports of successful radiofrequency ablation for ventricular tachycardia (VT), no large-scale studies have evaluated the role of VT/ventricular fibrillation (VF) ablation in cardiac amyloidosis [63]. Catheter ablation of VT has demonstrated some utility in patients with other infiltrative cardiomyopathies, such as cardiac sarcoidosis, suggesting it may be a viable option for patients with cardiac amyloidosis [64]. However, further large-scale studies are necessary to assess the risks and benefits of catheter ablation for ventricular tachycardia in cardiac amyloidosis, as current data do not show a mortality benefit. The development of refractory heart failure is typically observed with incessant VT and is associated with a poor prognosis.

## 5. Future Directions

As novel medical therapies enhance survival rates in cardiac amyloidosis, there is a growing need for prospective studies to address crucial questions. These include the superiority of rhythm control strategies, the optimal anticoagulation approach, predictors of lethal ventricular arrhythmias, and the mortality benefit of ICD placement. Ongoing research is essential to refine and individualize management strategies for arrhythmias and conduction disorders in this unique population.

Artificial intelligence (AI) and machine learning (ML) systems are increasingly being integrated into the management of cardiovascular diseases, providing significant advancements in diagnosis, risk stratification, and treatment planning. Similar to their application in coronary artery disease and atrial fibrillation, AI-based systems can play a crucial role in managing patients with amyloidosis, improving precision and outcomes [65]. The implementation of AI and ML in the management of amyloidosis can facilitate early detection, optimize treatment strategies, and predict patient outcomes more accurately, thus enhancing the overall quality of care [65].

## 6. Conclusions

The management of arrhythmias and conduction disorders in cardiac amyloidosis is a multifaceted challenge. Advancements in understanding their pathophysiology and epidemiology have been made, but the complex interplay of various factors demands ongoing research to refine therapeutic strategies. The evolving landscape of medical therapies and the increasing longevity of patients with cardiac amyloidosis underscore the necessity for a comprehensive and personalized approach to arrhythmia management in this unique population.

## Figures and Tables

**Table 1:** Epidemiology of cardiac amyloidosis.

Type of Amyloidosis	Prevalence	Key Characteristics	Demographics	Associated Risk Factors
wtATTR-CM	Increasing prevalence, especially in individuals over 80	Non-hereditary, amyloid deposits mainly in the heart	Predominantly affects elderly males	Age, male gender
hATTR-CM	Varies based on specific gene and geographic distribution	Hereditary, diverse clinical manifestations, including neuropathy and cardiomyopathy	All age groups, family history	Specific TTR gene mutations
AL Amyloidosis	Estimated 15.5 to 40.5 cases per million per year	Plasma cell proliferative disorder leading to systemic amyloid deposition, including in the heart and kidneys	Middle-aged and older adults, slightly more common in males	Underlying plasma cell dyscrasia, monoclonal light chains

AL amyloidosis: immunoglobulin light-chain amyloidosis; hATTR-CM: hereditary transthyretin cardiac amyloidosis; TTR: transthyretin; wtATTR-CM: wild-type transthyretin cardiac amyloidosis.

**Table 2:** Comparison of transthyretin and immunoglobulin light-chain amyloidosis.

Feature	wtATTR-CM	hATTR-CM	AL Amyloidosis
Genetic Basis	Non-hereditary	Hereditary; specific TTR gene variants	Plasma cell disorder producing monoclonal light chains
Clinical Manifestations	Predominantly cardiac; heart failure, arrhythmias	Variable based on variant; includes neuropathy, cardiomyopathy	Systemic; including kidneys, nerves, heart; heart failure, arrhythmias
Treatment Approaches	Tafamidis, heart transplantation	Gene-specific therapies, liver transplantation, Tafamidis	Chemotherapy, autologous stem cell transplantation, heart transplantation
Diagnostic Modalities	Echocardiography, cardiac MRI, scintigraphy	Genetic testing, echocardiography, cardiac MRI, scintigraphy	Serum and urine immunofixation, bone marrow biopsy, echocardiography, cardiac MRI
Prognostic Factors	Age of onset, cardiac involvement	Type of mutation, extent of organ involvement	Cardiac involvement, response to therapy, biomarkers (e.g., NT-proBNP)

AL Amyloidosis: immunoglobulin light-chain amyloidosis; hATTR-CM: hereditary transthyretin cardiac amyloidosis; MRI: magnetic resonance imaging; NT-proBNP: N-terminal pro-B-type natriuretic peptide; TTR: transthyretin; wtATTR-CM: wild-type transthyretin cardiac amyloidosis.

**Table 3:** Overview of arrhythmias and conduction anomalies in cardiac amyloidosis.

Type of Arrhythmia	Prevalence in Cardiac Amyloidosis	Clinical Impact	Management Considerations
Atrial Fibrillation	Higher than in the general population; up to 44% in wtATTR-CM	Increased thromboembolic risk, stroke	Anticoagulation (warfarin, NOACs), rate/rhythm control strategies
Ventricular Arrhythmias	Common; associated with sudden death risk	Life-threatening, impacts quality of life	ICD for secondary prevention, antiarrhythmic drugs, ablation in select cases
Supraventricular Tachycardias	Not well quantified	Can exacerbate heart failure symptoms	Rate control, potentially rhythm control (e.g., ablation)
Intrinsic Conduction Disease	Prevalence data varies; significant in advanced stages	Leads to bradycardia, heart block	Pacemaker implantation for symptomatic bradycardia or advanced heart block

ICD: implantable cardioverter-defibrillator; NOACs: novel oral anticoagulants; wtATTR-CM: wild-type transthyretin cardiac amyloidosis.

**Table 4:** Management strategies for arrhythmias in cardiac amyloidosis.

Treatment Strategy	Indications	Mechanism of Action	Benefits	Limitations and Considerations
Pharmacotherapy (e.g., Amiodarone, Beta-Blockers)	Rate and rhythm control in atrial arrhythmias	Varied mechanisms depending on drug class	Effective in managing arrhythmias, improving symptoms	Potential side effects, monitoring required, may exacerbate heart failure
Rate Control (e.g., Beta-Blockers, CCBs)	Managing rapid ventricular rates in AF and other SVTs	Reducing atrio-ventricular nodal conduction	Reduces symptoms, prevents tachycardia-induced cardiomyopathy	Caution in patients with advanced heart failure, potential side effects, CCBs contraindicated
Rhythm Control (e.g., DCCV, Antiarrhythmic Drugs)	Restoration and maintenance of sinus rhythm	Conversion to sinus rhythm (DCCV), altering cardiac electrical properties (drugs)	Can improve hemodynamics and symptoms	Risk of recurrence, procedural risks, drug side effects
ICD Implantation	Secondary prevention of ventricular arrhythmias and sudden death	Automatic detection and termination of life-threatening arrhythmias	Can prevent sudden cardiac death	Limited data on efficacy in primary prevention, procedural risks

AF: atrial fibrillation; CCBs: calcium channel blockers; DCCV: direct current cardioversion; ICD: implantable cardioverter-defibrillator; SVTs: supraventricular tachycardias.

## Data Availability

Not applicable.
